# Social cohesion, mental wellbeing and health-related quality of life among a cohort of social housing residents in Cornwall: a cross sectional study

**DOI:** 10.1186/s12889-020-09078-6

**Published:** 2020-06-22

**Authors:** Andrew James Williams, Kath Maguire, Karyn Morrissey, Tim Taylor, Katrina Wyatt

**Affiliations:** 1 Division of Population and Behavioural Science, School of Medicine, University of St Andrews, St Andrews, Fife, KY16 9TF UK; 2 European Centre for Environment and Human Health, University of Exeter Medical School, Knowledge Spa, Royal Cornwall Hospital, Truro, Cornwall, TR1 3HD UK; 3 University of Exeter Medical School, Exeter, Devon, UK

**Keywords:** Social cohesion, Mental wellbeing, Health, Social housing, Social capital

## Abstract

**Background:**

Research and policy have identified social cohesion as a potentially modifiable determinant of health and wellbeing that could contribute to more sustainable development. However, the function of social cohesion appears to vary between communities. The aim of this study was to analyse the levels of, and associations, between social cohesion, mental wellbeing, and physical and mental health-related quality of life among a cohort of social housing residents from low socioeconomic status communities in Cornwall, UK. Social housing is below market-rate rental accommodation made available to those in certain health or economic circumstances. These circumstances may impact on the form and function of social cohesion.

**Methods:**

During recruitment, participants in the Smartline project completed the Short Warwick-Edinburgh Mental Wellbeing Scale, SF-12v2 and an eight item social cohesion scale. Cross sectional regression analyses of these data adjusted for gender, age, national identity, area socioeconomic status, rurality, education, employment, and household size were undertaken to address the study aim.

**Results:**

Complete data were available from 305 (92.7%) participants in the Smartline project. Univariable analyses identified a significant association between social cohesion, mental wellbeing and mental health-related quality of life. Within fully adjusted multivariable models, social cohesion only remained significantly associated with mental wellbeing. Sensitivity analyses additionally adjusting for ethnicity and duration of residence, where there was greater missing data, did not alter the findings.

**Conclusions:**

Among a relatively homogeneous cohort, the reported level of social cohesion was only found to be significantly associated with higher mental wellbeing, not physical or mental health-related quality of life. The efforts made by social housing providers to offer social opportunities to all their residents regardless of individual physical or mental health state may support the development of a certain degree of social cohesion. Sense of control or safety in communities may be more critical to health than social cohesion. Additional observational research is needed before attempts are made to alter social cohesion to improve health.

## Background

Empirical research has identified associations between social cohesion and a wide variety of health states and health-related behaviours; all-cause mortality [1], preventive healthcare use [2], mental health [3, 4], smoking [5], alcohol consumption [6], sexual health [6], physical activity [7], obesity [8], socioeconomic inequalities [9, 10] and antisocial behaviours [7, 11, 12]. However, the meaning of social cohesion is debated. Carrasco and Bilal [13] discussed the conflation of the concepts of social cohesion and social capital, as demonstrated by the World Health Organization definition [14]. Instead Carrasco and Bilal [13] state that while capital is something that an individual or community might have, cohesive is something that a community might be [13]. They conclude that ‘the practical implication of focusing on “having” vs. “being” includes an emphasis on understanding how to normalize groups and populations rather than providing those groups space for empowerment and agency leading to health.’ ([13], p.127)

Primarily conceptualised as a determinant of health, social cohesion has also been discussed as being determined by health [14–17]. Therefore, social cohesion has been seen as both an objective in itself, as well as a process by which to improve the health of the public. It is understandable that enhancing social cohesion has been referenced as a means of improving health in many policies (e.g. [18, 19]). However, the evidence published more recently has begun to reveal the need for social cohesion to be studied at a local level, as the function of social cohesion appears to vary by the characteristics of the community being studied [3, 6]. In two US communities, Walton [3] found that homeowners and white people experienced mental health benefits associated with their sense of community, which renters and people of colour in the same communities did not experience. In Mexico, Lozano et al. [5] found social cohesion to modify the influence of social norms in regards to smoking. While, Lippman et al. [6] found an association between higher social cohesion and HIV prevention behaviours in one South African community, but not another which they hypothesised was caused by contextual differences in the communities such as diversity, geography and history. White et al. [20] measured social cohesion as one of the secondary outcomes (after mental health) in their natural experimental evaluation of neighbourhood regeneration projects in South Wales, UK. They found that a neighbourhood regeneration programme (Communities First) which targeted the 100 most socioeconomically deprived (out of 881) electoral wards in Wales was associated with improvements in mental health, potentially narrowing mental health inequalities (intervention *n* = 4197, control *n* = 6695) [20]. However, the changes they observed in social cohesion were similar within the intervention and propensity score matched control groups, with the percentage of people reporting medium-levels of social cohesion reducing and the percentage reporting low and high social cohesion increasing [20]. These examples of heterogeneity in the associations between social cohesion, health and wellbeing suggest that it is an ecological fallacy to assume that the associations between social cohesion and health observed at the national level are true at the local level. Communities can be defined in multiple ways (e.g. place, interest, circumstance, identity) which rarely correspond with administrative geographies used by governments. Extending the argument of Carrasco and Bilal [13] it is important to study social cohesion in communities who are defined by an attribute around which they might cohere.

The Smartline project is a three-year research project in a European region of low economic output (Cornwall, UK), funded by the European Region Development Fund. The project is a collaboration between the University of Exeter, Coastline Housing (a social housing provider, whose customers were invited to participate in the project), Volunteer Cornwall (a charity which develops individuals and communities through voluntary action) and Cornwall Council (the local government) [21]. The objective of the Smartline project was to explore and test the opportunities for technology to support people to live more healthily and happily in their homes and communities [21]. The 2016 to 17 English Housing Survey reported that 2.4 million households were renting from a social housing provider [22]. Social housing providers are private not-for-profit organisations who provide rental properties at around 50–60% of market rates for those whose health or economic circumstances exclude them from the private market [22–24]. The role of social housing providers in the UK has evolved over recent decades to include supporting the social engagement of their residents and communities [25, 26]. Consequently, the Smartline project offered the opportunity to study an important evidence gap regarding the potential role of social cohesion among communities that were not just defined geographically, but also by their shared circumstances, which include opportunities to become involved with their neighbourhood that may not be available to private renters or owner occupiers in the same neighbourhoods. The overall aim of the present study was to describe the levels of social cohesion among these social housing residents and examine how this was associated with socio-demographic characteristics, mental wellbeing and health-related quality of life. We sought to test the hypothesis that the associations identified among the Smartline households would be different from those identified in wider population studies. Thus in turn contributing to a more nuanced understanding of the role of social cohesion at the local level in improving the health of the public.

## Methods

All the participants in the Smartline project were recruited from the towns and villages of Camborne, Pool, Illogan and Redruth in West Cornwall, UK. This represents the largest urban conurbation in Cornwall, with 11% of the Cornish population [27]. This area was selected as it contains the highest concentration of Coastline homes, as the project required participants living close to each other in order to be able to study communities as well as individuals. A number of additional features of the study location contribute to the importance of studying social cohesion in these communities. Firstly, Cornwall is located on a peninsula and only borders one other county in England. The Camborne, Pool, Illogan and Redruth area lies in the heart of the county, distant from the Cornish beaches that provide the base for the county’s tourism industry. Historically this area was dominated by mining, but the area suffered during the gradual decline of the industry, with the last mine closing in 1998. This area faces significant geographic barriers, being distant from major cities (with Plymouth and Exeter being at least 80 min travel away).

The Smartline project sought to recruit 350 homes to the 3 year project. Coastline Housing undertook the recruitment street by street, contacting every one of their residents on each street and visiting those who responded positively to collect consent, before moving onto the next street until approximately 350 households had been recruited. In total they approached 649 households; of whom 329 agreed to participate (50.7% response rate). At the beginning of the project a survey was undertaken with each of the participating households to gather data on the household (number of rooms, heating practices, etc.) and health and wellbeing of the main participant (required to be an adult ≥18 years of age). All the surveys took place in the participant’s home at a convenient time of the day (usually between 9 am and 5 pm) with two researchers present, after the participant had given written informed consent. Data collection took place between September 2017 and April 2018 (*n* = 303) with a booster sample undertaken between August and November 2018 (*n* = 26). The Smartline project was approved by the University of Exeter Research Ethics Committee and conformed to the principles embodied in the Declaration of Helsinki.

Social cohesion was identified by the investigators and project partners as a potentially modifiable determinant of health and wellbeing that could be affected by technology. While social cohesion has been the focus of much research, there has been little consistency in how it has been measured with respondents asked to rate a series of between four and thirteen statements on community connectivity and trust using a 5-point Likert scale [2–4, 6, 7, 11, 12, 28], but elsewhere census derived measures have been used [29].The 8-item social cohesion questionnaire developed by White et al. [28] from Buckner’s Neighbourhood Cohesion Scale [30] was included in the survey. The eight statements relate to relationships with friends and neighbours, and activities such as visiting, helping in an emergency, borrowing and exchanging favours, and the participant rated each statement from ‘strongly agree’ to ‘strongly disagree’. These social cohesion data alongside sociodemographic data (using questions based on the English Census 2011 [31]) and mental wellbeing and health-related quality of life data (listed in Table 1) were analysed to address the current studies aim. Mental wellbeing was assessed using the validated and widely used Short Warwick-Edinburgh Mental Wellbeing Scale (SWEMWBS), which ‘was developed to enable the monitoring of mental wellbeing in the general population and the evaluation of projects, programmes and policies which aim to improve mental wellbeing.’ [32–36] The developers defined mental wellbeing as the ‘positive aspect of mental health’, using the World Health Organization definition of mental health: ‘a state of well-being in which an individual realizes his or her own abilities, can cope with the normal stresses of life, can work productively and is able to make a contribution to his or her community.’ [32, 37] The SF-12v2 Health Survey was used to collect data on the participants state of health [38, 39]. SF-12v2 is a:

‘multipurpose, short-form health survey with 12 questions that yields an eight-scale [physical functioning, role physical, bodily pain, general health, vitality, social functioning, role emotional and mental health] profile of functional health and well-being, as well as two psychometrically based physical and mental health summary measures [physical and mental component summaries] and a preference-based health utility index.’ (p.3) [38]

For this study we used the physical and mental component summaries (PCS and MCS) of the SF-12v2 which respectively are measures of physical and mental (and psychological) morbidity and aetiology especially in relation to impact on functioning and therefore within this paper we refer to them as measures of physical and mental health-related quality of life [38].

SWEMWBS, SF12-v2 and the social cohesion questionnaire were coded using standard protocols [32, 38, 39]. Where more than two responses were missing from either the 8-item social cohesion measure, or the 7-item SWEMWBS the total score was classified as missing, otherwise the mean of the other responses was used in place of the 1 or 2 missing values. For SF-12v2 missing score estimation was implemented within the Health Outcomes Scoring software 5.1 [38, 39]. Index of Multiple Deprivation (IMD 2015) and Rural Urban classification (2011, RUC11) of the home postcode were linked to the survey data [40, 41]. Coastline Housing, as the landlord, provided details on how long each participant had been resident in that property. Sociodemographic variables were coded as categorical variables, with categories being combined when there was a risk of disclosing someone’s identity. The categories used throughout the analysis are show in Table 1.

The analysis took a cross-sectional complete case approach. Although the intention had been to include ethnicity and duration of residence in the analysis, there were larger quantities of missing data (34.7% of participants (*n* = 114)) for these variables and therefore they were reserved for a sensitivity analysis. Following cleaning and coding of the data, each variable was summarised to characterise the participants and comparisons were made between those with and without complete data and compared to local or national data to assess biases which would need to be considered [42]. SWEMWBS national comparison data was taken from the Health Survey for England 2011 which are recommended as the UK population norms, while the Welsh Health Survey 2015 was the national comparison for SF-12v2 Health Survey [43, 44].

Univariable linear regression models were used to assess the unadjusted associations between each of the proposed explanatory participant characteristic variables and social cohesion, SWEMWBS, SF-12v2 Health Survey physical (PCS) and mental component summaries (MCS). Finally, multivariable linear regression models of SWEMWBS, PCS and MCS were estimated with the proposed explanatory variables with and without adjustment for social cohesion to assess the adjusted association between social cohesion and health-related quality of life and wellbeing. These final models were repeated adding ethnicity and duration of residence as a sensitivity analysis. All analyses were undertaken in Stata [45] using two-tailed tests and a sensitivity value of 0.05.

## Results

Of the 329 Smartline participants, complete data for the variables of interest in this study was available for 308 (93.6%). All but three participants of the Smartline project lived in the 40% most deprived postcodes in England, and consequently to avoid identifying these three people they were added to the group with missing data. With the exception of ethnicity, socioeconomic status and education, the complete cases and those with missing data did not differ statistically significantly. Table 1 summarises the characteristics of the whole sample of participants also stratified by level of social cohesion [20].

**Table 1:** Participant characteristics for the complete sample and by level of social cohesion

		Complete (n-305)^a^	Social cohesion^b^			
			Low (*n* = 20)^a^	Medium (*n* = 212)^a^	High (*n* = 73)^a^	p^c^
Age	Years	54.1 ± 17.6	55.7 ± 19.8	53.6 ± 17.4	55.0 ± 17.8	0.78
Gender	Male	31.2%	20.0%	35.4%	21.9%	0.05
	Female	68.9%	80.0%	64.6%	78.1%	
Ethnicity	White	97.5%	100.0%	97.6%	96.6%	0.77
	Other	2.5%	0.0%	2.4%	3.4%	
National identity	Cornish	47.5%	55.0%	49.5%	39.7%	0.41
	British	35.4%	35.0%	32.6%	43.8%	
	Other	17.1%	10.0%	17.9%	16.4%	
IMD 2015	ranking	4446 (964–8548)	4518.5 (1048.5–10,826)	4446 (964–8548)	1512 (964–8548)	0.58
	1st decile (most)	49.2%	40.0%	48.6%	53.4%	0.84
	2nd decile	15.4%	15.0%	16.0%	13.7%	
	3rd decile	12.5%	10.0%	12.3%	13.7%	
	4th decile	23.0%	35.0%	23.1%	19.2%	
	5th decile	0.0%	0.0%	0.0%	0.0%	
	6th decile	0.0%	0.0%	0.0%	0.0%	
	7th decile	0.0%	0.0%	0.0%	0.0%	
	8th decile	0.0%	0.0%	0.0%	0.0%	
	9th decile	0.0%	0.0%	0.0%	0.0%	
	10th decile (least)	0.0%	0.0%	0.0%	0.0%	
RUC 2011	Urban city and town	93.1%	85.0%	94.8%	90.4%	0.15
	Other	6.9%	15.0%	5.2%	9.6%	
Education	Secondary and/or primary	66.2%	60.0%	65.6%	69.9%	0.31
	Further	28.5%	40.0%	29.7%	21.9%	
	Higher	5.3%	0.0%	4.7%	8.2%	
Employment	In work	19.7%	10.0%	20.3%	20.6%	0.57
	Education or training	2.3%	0.0%	2.4%	2.7%	
	Retired	33.4%	30.0%	31.6%	39.7%	
	Not in work	44.6%	60.0%	45.8%	37.0%	
Household size	1	39.6%	50.0%	41.5%	31.5%	0.26
	2	30.5%	45.0%	27.4%	35.6%	
	3	14.4%	0.0%	15.1%	16.4%	
	4	10.5%	0.0%	11.3%	11.0%	
	5+	4.9%	5.0%	4.7%	5.5%	
Duration of residence	< 1 year	13.3%	12.5%	14.7%	9.8%	0.05
	1–3 years	25.4%	6.25%	25.2%	31.2%	
	4–6 years	23.8%	56.3%	19.0%	27.9%	
	7–9 years	9.6%	12.5%	9.8%	8.2%	
	≥10 years	27.9%	12.5%	31.3%	23.0%	
Social cohesion		26.9 ± 6.5	13.1 ± 2.6	25.4 ± 4.0	34.8 ± 2.4	–
Mental wellbeing (SWEMWBS)		24.1 ± 5.2	20.5 ± 5.1	23.9 ± 5.1	25.8 ± 4.9	< 0.01
Physical HRQoL (SF-12v2 PCS)		41.7 (28.6–53.4)	40.9 (26.5–50.7)	41.7 (29.0–53.6)	41.2 (27.8–54.3)	0.70
Mental HRQoL (SF-12v2 MCS)		52.3 (39.0–58.4)	39.3 (28.9–54.9)	52.2 (41.0–58.2)	52.5 (38.9–59.7)	0.12

*HRQoL* Health-Related Quality of Life, *IMD 2015* Index of Multiple Deprivation [41], *MCS* Mental component summary of SF-12v2 Health Survey, *PCS* Physical component summary of SF-12v2 Health Survey, *RUC 2011* Rural Urban Classification 2011 [40], *SWEMWBS* Short Warwick-Edinburgh Mental Wellbeing Scale [32], *SF-12v2* SF-12v2 Health Survey [38, 39]

^a^ Values present as mean ± standard deviation where data were normally distributed, median (interquartile range) when data were skewed and percentages for categorical data

^b^ Cut points for social cohesion copied from White et al. [20] with low = 0–16; medium = 17–31; high = 32–40

^c^Tests comparing level of social cohesion groups using one way ANOVA, chi-squared or Kruskal-Wallis equality-of-populations rank tests

**Fig. 1:**
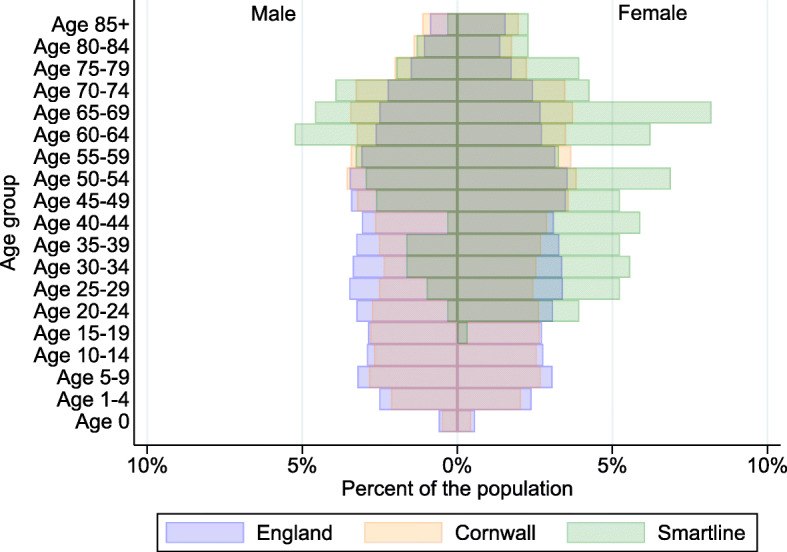
Population pyramid comparing the complete cases from the Smartline project with the mid-2017 population estimates for the county of Cornwall and country of England. Legend: Source: Office for National Statistics Population Estimates for UK, England and Wales, Scotland and Northern Ireland: Mid-2017 [42]

While Cornwall is typically considered a rural county, 93.1% of the homes were classified as being in an urban city or town. Approximately a third of participants had undertaken any further or higher education and only 22.0% were in employment, education or training (33.4% had already retired). Most participating households had only 1–2 people residing there, with most people having lived in that home for 1–6 years, although 27.9% of participants had lived in their home for 10 or more years. Compared to the Office for National Statistics mid-2017 population estimates for England as a whole and Cornwall the county, the sample analysed in this study was older and had a higher proportion of females (Fig. 1) [42].

The SWEMWBS, PCS, and MCS were similar to the national comparators from England and Wales. The mean and standard deviation of the SWEMWBS scores from the 2011 Health Survey for England, was 23.6 ± 3.9, where these figures for the Smartline complete cases were 24.1 ± 5.2 [32, 36]. The PCS and MCS of SF-12v2 of the Smartline complete cases were not normally distributed and hence medians and interquartile ranges were compared with these values from the 2015 Welsh Health Survey [43]. The median (interquartile range) MCS scores of the Smartline complete cases and Welsh Health Survey 2015 were respectively; 52.3 (39.0–58.4) and 52.1 (43.7–57.2). While for the PCS the difference was more marked, but not statistically significant, Smartline; 41.7 (28.6–53.4), Welsh Health Survey 2015; 53.5 (42.8–59.9).

No national surveys of England or the UK have used the same social cohesion questionnaire used in current study, however, White et al. [20] categorised low, medium and high levels of social cohesion using the same questionnaire among their participant’s with 25.8% reporting low, 36.6% medium and 37.6% high social cohesion at baseline. Applying the same categorisation to the Smartline complete cases, 6.6% reported low, 69.5% medium and 23.9% high social cohesion (Table 1). Neighbourhood cohesion data are collected as part of the UK Household Longitudinal Study (UKHLS, Understanding Society) using a 13-item questionnaire [4]. Papachristou et al. [4] examined the associations between neighbourhood cohesion, inflammation and psychological distress among a cross-section of Understanding Society. They reported that the mean (standard deviation) neighbourhood cohesion scores out of a possible 13 among their analytical sample (*n* = 9393) was 11.54 (2.14) and among the non-analytical sample (*n* = 27,393) it was 11.25 (2.36) [4]. The low levels of completion of the neighbourhood cohesion questionnaire caution against weighting these data to provide nationally representative data [4, 46]. However, comparing the means from the Smartline and Understanding Society cohorts as percentages of the highest possible scores for each questionnaire, the Understanding Society mean was over 85% of the highest possible score, while for the Smartline cohort this was only 67% [4]. While different questionnaires were used and the samples were each biased in different ways, it appears as though the levels of cohesion reported by the Smartline cohort were markedly lower than those found in Understanding Society. Across all the variables analysed only SWEMWBS score was found to statistically significantly vary between levels of social cohesion (Hedges’ g effect sizes: low vs. medium 0.65, medium vs. high 0.37, low vs. high 1.03), with borderline significant variation in gender (men most likely to report medium levels of social cohesion) and duration of residence (Table 1).

The univariable analyses (Table 2) found that only mental wellbeing (SWEMWBS) and mental health-related quality of life (MCS) were significantly positively associated with social cohesion. The variables found to be significantly associated with mental wellbeing and mental health-related quality of life were similar with increasing age, employment and social cohesion being positively associated. Whereas, not living in the 10% most deprived postcodes in England was found to be associated with poorer mental health-related quality of life, and increasing levels of education were associated with poorer mental wellbeing among this group. Higher physical health-related quality of life (PCS) was significantly associated with, greater duration of education, being in employment, education or training compared to not working, and living in larger households. Lower physical health-related quality of life was significantly associated with aging, living in an urban area and being retired although this is, of course, related to age. Physical health-related quality of life was not significantly associated with social cohesion, mental wellbeing or mental health-related quality of life in the univariable analyses.

**Table 2:** Univariable regression models of associations between participant characteristics and social cohesion, mental wellbeing, physical and mental health related quality of life

		Social Cohesion		Mental wellbeing (SWEMWBS)		Physical health-related quality of life (SF-12v2 PCS)		Mental health-related quality of life (SF-12v2 MCS)	
		Coef	95% CI	Coef	95% CI	Coef	95% CI	Coef	95% CI
Age	Years	< 0.01	−0.04 to 0.04	**0.08**	**0.04 to 0.11**	**−0.30**	**− 0.38 to − 0.22**	**0.17**	**0.08 to 0.25**
Gender	Female	ref		ref		ref		ref	
	Male	−0.77	− 2.34 to 0.80	0.02	−1.26 to 1.29	−3.04	−6.40 to 0.31	2.66	−0.64 to 5.96
Ethnicity	White	ref		ref		ref		ref	
	Other	0.88	−4.16 to 5.92	−3.83	−8.08 to 0.41	4.24	−7.04 to 15.52	2.51	−8.81 to 13.83
National identity	Cornish	ref		ref		ref		**ref**	
	British	0.99	−0.62 to 2.60	−0.69	−2.00 to 0.61	− 0.29	− 3.74 to 3.17	**− 1.70**	**−5.08 to 1.69**
	Other	1.90	−0.15 to 3.95	1.47	−0.19 to 3.13	3.36	−1.04 to 7.76	**5.14**	**−0.15 to 8.43**
IMD 2015	ranking	<−0.01	<−0.01 to < 0.01	<− 0.01	<− 0.01 to < 0.01	< 0.01	<− 0.01 to < 0.01	<−0.01	<− 0.01 to < 0.01
	1st decile (most)	ref		ref		ref		**ref**	
	2nd decile	−0.45	−2.57 to 1.68	−1.72	−3.44 to − 0.01	4.03	− 0.52 to 8.57	**−5.51**	**−9.94 to − 1.08**
	3rd decile	− 0.37	− 2.68 to 1.93	− 2.20	−4.06 to − 0.34	2.00	− 2.94 to 6.94	**−5.40**	**− 10.21 to − 0.59**
	4th decile	−1.30	−3.14 to 0.54	− 0.87	− 2.35 to 0.61	2.93	−1.00 to 6.87	**− 2.89**	**−6.72 to 0.95**
	5th decile	–	–	–	–	–	–	**–**	**–**
	6th decile	–	–	–	–	–	–	**–**	**–**
	7th decile	–	–	–	–	–	–	**–**	**–**
	8th decile	–	–	–	–	–	–	**–**	**–**
	9th decile	–	–	–	–	–	–	**–**	**–**
	10th decile (least)	–	–	–	–	–	–	**–**	**–**
RUC 2011	Urban city and town	ref		ref		**ref**		ref	
	Other	−0.01	−2.88 to 2.87	0.24	−2.10 to 2.58	**6.98**	**0.86 to 13.10**	−3.44	−9.48 to 2.61
Education	Secondary and/or primary	ref		**ref**		**ref**		ref	
	Further	−0.69	−2.32 to 0.94	**−1.86**	**−3.17 to − 0.54**	**5.84**	**2.42 to 9.27**	−0.88	−4.32 to 2.56
	Higher	1.48	−1.81 to 4.78	**−0.61**	**−3.27 to 2.04**	**7.66**	**−0.72 to 14.60**	0.58	−6.39 to 7.54
Employment	In work	1.05	−0.91 to 3.02	**1.48**	**−0.06 to 3.02**	**7.64**	**3.62 to 11.65**	**4.95**	**0.96 to 8.95**
	Education or training	1.95	−2.97 to 6.87	**0.45**	**−3.41 to 4.31**	**10.73**	**0.68 to 20.77**	**0.83**	**−9.17 to 10.82**
	Retired	1.27	−0.39 to 2.93	**3.38**	**2.08 to 4.69**	**−4.18**	**−7.58 to − 0.79**	**8.60**	**5.22 to 11.98**
	Not in work	ref		**ref**		**ref**		**ref**	
Household size	1	ref		ref		**ref**		ref	
	2	0.29	−1.45 to 2.03	−0.03	−1.47 to 1.40	**2.63**	**−0.93 to 6.19**	− 0.49	−4.19 to 3.22
	3	2.10	−0.12 to 4.32	0.64	−1.19 to 2.46	**8.14**	**3.59 to 12.69**	0.64	−4.09 to 5.37
	4	2.56	0.05 to 5.07	−0.19	−2.25 to 1.88	**11.55**	**6.42 to 16.69**	−2.44	−7.78 to 2.91
	5+	1.95	−1.50 to 5.41	0.02	−2.83 to 2.86	**15.17**	**8.10 to 22.24**	−0.20	−7.56 to 7.16
Duration of residence	< 1 year	−0.45	− 3.16 to 2.26	−1.28	−3.63 to 1.06	8.52	2.77 to 14.27	−7.56	−13.44 to − 1.69
	1–3 years	0.53	−1.70 to 2.77	−0.73	−2.66 to 1.20	3.16	−1.57 to 7.90	−2.70	−7.54 to 2.14
	4–6 years	−0.20	−2.47 to 2.07	−1.25	−3.21 to 0.72	1.33	−3.49 to 6.14	−1.76	−6.69 to 3.17
	7–9 years	−1.64	−4.69 to 1.40	− 1.03	−3.66 to 1.61	1.95	−4.51 to 8.41	−2.91	−9.52 to 3.70
	≥10 years	ref		ref		ref		ref	
Social cohesion		Dependant variable		**0.23**	**0.14 to 0.31**	0.03	−0.21 to 0.27	**0.30**	**0.07 to 0.54**
Mental wellbeing (SWEMWBS)		**0.34**	**0.21 to 0.47**	Dependant variable		0.07	−0.22 to 0.37	**1.64**	**1.41 to 1.87**
Physical HRQoL (SF-12v2 PCS)		0.01	−0.05 to 0.06	0.01	−0.03 to 0.05	Dependant variable		−0.06	−0.17 to 0.05
Mental HRQoL (SF-12v2 MCS)		**0.07**	**0.01 to 0.12**	**0.24**	**0.21 to 0.28**	−0.06	−0.18 to 0.05	Dependant variable	

95% CI; 95% confidence interval, Coef; regression coefficient, HRQoL; Health-Related Quality of Life, IMD 2015; Index of Multiple Deprivation 2015 [41], MCS; mental component summary of SF-12v2 Health Survey, PCS; physical component summary of SF-12v2 Health Survey, RUC 2011; Rural Urban Classification 2011 [40], SWEMWBS; Short Warwick-Edinburgh Mental Wellbeing Scale [32], SF-12v2; SF-12v2 Health Survey, [38, 39]

**Values in bold** indicate statistically significant (*p* < 0.05) variables

The multivariable regressions reported in Tables 3, 4 and 5 are consistent with the univariable analyses (Table 2). Adjusting for social cohesion only significantly improved the fit of the mental wellbeing model (Table 3). Whereas, adjusting for social cohesion did not significantly improve the fit of the models of physical or mental health-related quality of life (Tables 4 and 5). At most, the fitted explanatory variables explained around 15% of the variation in mental wellbeing, 19% of the variation in physical health-related quality of life and 10% of the variation in mental health-related quality of life. The sensitivity analyses presented in the additional file (Tables S1-S3) are consistent with the main analysis. Neither ethnicity, nor duration of residence were consistently statistically significantly associated with mental wellbeing, or physical or mental health-related quality of life. Notably, there was no evidence of a dose response relationship between duration of residence and any of the outcomes. Tests were undertaken to ensure that none of the assumptions of linear regression were breached, especially in relation to the distribution of PCS and MCS and no breaches were observed.

**Table 3:** Adjusted regression of mental wellbeing (Short Warwick-Edinburgh Mental Wellbeing Scale, SWEMWBS) (*n* = 305) [32]

		Unadjusted		Adjusted for SC	
		Coef	95% CI	Coef	95% CI
Intercept		**19.38**	**15.98 to 22.77**	**14.68**	**10.70 to 18.67**
Age	Years	**0.06**	**0.01 to 0.12**	**0.06**	**0.01 to 0.12**
Gender	Female	ref		ref	
	Male	−0.21	−1.48 to 1.05	− 0.13	− 1.36 to 1.10
National identity	Cornish	ref		ref	
	British	0.15	−1.17 to 1.47	−0.08	− 1.37 to 1.20
	Other	1.85	0.20 to 3.50	1.46	−0.16 to 3.07
IMD 2015	1st decile (most)	ref		ref	
	2nd decile	−1.13	−2.85 to 0.58	−0.98	− 2.65 to 0.69
	3rd decile	−1.77	−3.64 to 0.10	−1.68	−3.50 to 0.14
	4th decile	−0.86	−2.52 to 0.79	− 0.55	− 2.17 to 1.06
	5th decile	–	–	–	–
	6th decile	–	–	–	–
	7th decile	–	–	–	–
	8th decile	–	–	–	–
	9th decile	–	–	–	–
	10th decile (least)	–	–	–	–
RUC 2011	Urban city and town	ref		ref	
	Other	0.17	−2.47 to 2.80	0.03	−2.53 to 2.59
Education	Secondary and/or primary	ref		ref	
	Further	−0.88	−2.31 to 0.55	−0.69	−2.09 to 0.70
	Higher	−0.36	−3.15 to 2.44	−0.44	−3.16 to 2.28
Employment	In work	1.10	−0.51 to 2.70	0.97	−0.59 to 2.54
	Education or training	1.08	−2.95 to 5.11	0.88	−3.04 to 4.81
	Retired	1.90	0.06 to 3.75	1.53	−0.27 to 3.34
	Not in work	ref		ref	
Household size	1	ref		ref	
	2	0.94	−0.51 to 2.39	0.86	−0.55 to 2.27
	3	2.48	0.54 to 4.42	2.02	0.13 to 3.92
	4	2.27	−0.03 to 4.57	1.69	−0.57 to 3.94
	5+	3.06	0.09 to 6.03	2.51	−0.39 to 5.40
Social cohesion		–	–	**0.19**	**0.10 to 0.27**

*IMD 2015* Index of Multiple Deprivation 2015 [41], *RUC 2011* Rural Urban Classification 2011 [40], *SC* Social cohesion

Likelihood ratio test *p* < 0.0001, Adjusted R^2^ of the unadjusted model; 0.0984, and adjusted model; 0.1466

**Values in bold** indicate statistically significant (*p* < 0.05) variables

**Table 4:** Adjusted regression of physical health-related quality of life (SF-12v2 Health Survey Physical component summary) (n = 305) [38, 39]

		Unadjusted		Adjusted for SC	
		Coef	95% CI	Coef	95% CI
Intercept		**51.97**	**43.48 to 60.47**	**53.38**	**43.11 to 63.34**
Age	Years	**−0.29**	**− 0.43 to − 0.15**	**−0.29**	**− 0.43 to − 0.15**
Gender	Female	ref		ref	
	Male	−0.85	−4.01 to 2.31	− 0.87	−4.04 to 2.29
National identity	Cornish	ref		ref	
	British	−2.17	−5.47 to 1.12	−2.11	−5.42 to 1.21
	Other	1.68	−2.45 to 5.81	1.79	−2.37 to 5.96
IMD 2015	1st decile (most)	ref		ref	
	2nd decile	0.68	−3.62 to 4.98	0.64	− 3.67 to 4.95
	3rd decile	0.29	−4.39 to 4.97	0.26	−4.43 to 4.95
	4th decile	−0.73	−4.88 to 3.42	−0.82	−4.99 to 3.35
	5th decile	–	–	–	–
	6th decile	–	–	–	–
	7th decile	–	–	–	–
	8th decile	–	–	–	–
	9th decile	–	–	–	–
	10th decile (least)	–	–	–	–
RUC 2011	Urban city and town	ref		ref	
	Other	5.91	−0.68 to 12.50	5.95	−0.65 to 12.55
Education	Secondary and/or primary	ref		ref	
	Further	0.95	−2.63 to 4.53	0.89	−2.70 to 4.49
	Higher	1.13	−5.86 to 8.12	1.15	−5.84 to 8.15
Employment	In work	**6.70**	**2.68 to 10.72**	**6.74**	**2.71 to 10.77**
	Education or training	**7.76**	**−2.33 to 17.84**	**7.81**	**−2.29 to 17.92**
	Retired	**4.94**	**0.32 to 9.56**	**5.05**	**0.40 to 9.70**
	Not in work	**ref**		**ref**	
Household size	1	ref		ref	
	2	0.68	−2.94 to 4.30	0.70	−2.93 to 4.33
	3	2.83	−2.02 to 7.67	2.96	−1.92 to 7.85
	4	2.44	−3.32 to 8.20	2.61	−3.20 to 8.42
	5+	8.59	1.16 to 16.02	8.75	1.28 to 16.22
Social cohesion		–	–	−0.06	−0.28 to 0.17

*IMD 2015* Index of Multiple Deprivation 2015 [41], *RUC 2011* Rural Urban Classification 2011 [40], *SC* Social cohesion

Likelihood ratio test *p* = 0.6197, Adjusted R^2^ of the unadjusted model; 0.1881, and adjusted model; 0.1859

**Values in bold** indicate statistically significant (*p* < 0.05) variables

**Table 5:** Adjusted regression of mental health-related quality of life (SF-12v2 Health Survey Mental component summary) (n = 305) [38, 39]

		Unadjusted		Adjusted for SC	
		Coef	95% CI	Coef	95% CI
Intercept		**36.63**	**27.80 to 45.45**	**31.14**	**20.53 to 41.74**
Age	Years	0.11	−0.03 to 0.26	0.12	−0.03 to 0.26
Gender	Female	ref		ref	
	Male	2.12	−1.16 to 5.41	2.22	−1.06 to 5.49
National identity	Cornish	ref		ref	
	British	−0.02	−3.44 to 3.41	−0.29	−3.71 to 3.14
	Other	4.65	0.36 to 8.94	4.19	−0.11 to 8.50
IMD 2015	1st decile (most)	ref		ref	
	2nd decile	−5.19	−9.66 to −0.72	−5.01	−9.46 to − 0.56
	3rd decile	−3.84	−8.71 to 1.02	−3.73	−8.58 to 1.11
	4th decile	−1.82	−6.13 to 2.49	−1.46	−5.77 to 2.85
	5th decile	–	–	–	–
	6th decile	–	–	–	–
	7th decile	–	–	–	–
	8th decile	–	–	–	–
	9th decile	–	–	–	–
	10th decile (least)	–	–	–	–
RUC 2011	Urban city and town	ref		ref	
	Other	−4.59	−11.44 to 2.26	−2.75	−11.58 to 2.07
Education	Secondary and/or primary	ref		ref	
	Further	2.18	−1.54 to 5.90	2.40	−1.31 to 6.11
	Higher	3.19	−4.07 to 10.45	3.09	−4.14 to 10.32
Employment	In work	**4.13**	**−0.05 to 8.31**	3.98	−0.18 to 8.15
	Education or training	**1.69**	**−8.79 to 12.18**	1.47	−8.97 to 11.91
	Retired	**6.16**	**1.36 to 10.96**	5.73	0.93 to 10.53
	Not in work	**ref**		ref	
Household size	1	ref		ref	
	2	2.24	−1.53 to 6.00	2.14	−1.60 to 5.89
	3	4.73	−0.31 to 9.77	4.20	−0.85 to 9.25
	4	2.60	−3.38 to 8.58	1.92	−4.09 to 7.92
	5+	7.82	0.10 to 15.54	7.17	−0.55 to 14.89
Social cohesion		–	–	0.22	−0.02 to 0.45

*IMD 2015* Index of Multiple Deprivation 2015 [41], *RUC 2011* Rural Urban Classification 2011 [40], *SC* Social cohesion

Likelihood ratio test *p* = 0.0606, Adjusted R^2^ of the unadjusted model; 0.0905, and adjusted model; 0.0978

**Values in bold** indicate statistically significant (*p* < 0.05) variables

## Discussion

Despite socioeconomic circumstances, the levels of mental wellbeing and mental and physical health-related quality of life reported among Smartline participants were not statistically significantly different to those reported from national health surveys. The greatest difference was in physical health-related quality of life; however the age profile of Smartline participants and the prioritised allocation of social housing to those with health conditions are likely to explain this difference [24]. Although the diversity of questionnaires for assessing social cohesion prevent direct comparison, the levels of social cohesion among the Smartline cohort appear to be lower than those reported in a large UK study (Understanding Society) [4]. However, Understanding Society is a representative survey of the UK, while the Smartline cohort are much more homogenous especially in terms of socioeconomic status and housing provider, which may explain the observed difference [46]. When compared to a community cohort in Wales with a similar socioeconomic demographic, the levels of cohesion were broadly similar although more of the Smartline cohort reported medium levels of social cohesion, and fewer reported high or low levels of social cohesion. Having adjusted for gender, age, national identity, index of multiple deprivation, rurality, education, employment and household size, social cohesion was only found to be significantly associated with mental wellbeing (Tables 3, 4 and 5). As a cross-sectional study it is not possible to identify whether social cohesion leads to improved mental wellbeing, or vice versa. No statistically significant association was found between social cohesion and physical or mental health-related quality of life (Tables 2, 3, 4 and 5). None of the sociodemographic characteristics that were adjusted for in the model were statistically significantly associated with social cohesion (Tables 3, 4 and 5). All of which supports our hypothesis that the associations found between health and wellbeing and social cohesion among a cohort of social housing residents in similar socioeconomic circumstances differed from those observed in larger or national studies [1, 3, 4].

All participants in the Smartline project were residents with the same social housing provider (Coastline Housing). This may explain the lack of associations between sociodemographic factors and social cohesion, as opportunities to develop a sense of community are being actively provided by Coastline Housing [25, 26]. The Coastline Housing Offices are located within the Camborne, Pool, Illogan and Redruth area which affords them specific local knowledge and the ability to offer local opportunities to the residents. The lack of a statistically significant association between physical health-related quality of life and social cohesion challenges the notion that not only is good social cohesion conducive to physical health, but poor physical health is detrimental to social cohesion [15, 17]. While there was evidence of socioeconomic inequalities in health-related quality of life and mental wellbeing among the Smartline participants, these did not extend to social cohesion (Tables 2, 3, 4 and 5). This finding is contrary to the hypothesis that social cohesion is a protective factor against socioeconomic inequalities [9, 10] but it is consistent with the finding of the Communities First study that social cohesion did not change in a way that would explain the mental health improvements resulting from the neighbourhood regeneration programme [28]. The social norm hypothesis [47] may explain the finding that mental health-related quality of life was poorer among the less deprived participants. However exploratory posthoc analyses failed to find any evidence that the social norm hypothesis explained the findings in this study and therefore additional exploration is needed. The opportunities offered by social housing providers may intentionally overcome physical or mental health related barriers to residents’ community engagement [25, 26].

A greater proportion of Smartline participants reported medium social cohesion compared with the similar cohort of participants in the Communities First study from Caerphilly county borough in south Wales [20] although the full range of possible social cohesion scores were reported by the Smartline participants. Which, alongside the similarity to the nationally-representative data on mental wellbeing and, mental and physical health-related quality of life, indicates that the absence of some of the previously identified associations with social cohesion are unlikely to be due to lower variation in the outcomes among the Smartline cohort. Other studies have found greater associations between social cohesion and mental wellbeing and mental health-related quality of life than physical health-related quality of life [3]. While studies by Walton [3] and Lippman et al. [6] found communities in which social cohesion was not associated with mental health or health-related behaviours (smoking). Both Lozano et al. [5] and Walton [3] identified contextual variables such as renting and social norms as having an impact on the potential for greater social cohesion to impact on health.

The Smartline participants had fairly uniform sociodemographic characteristics and low ethnic diversity, which although it limits external validity, is valuable in the study of social cohesion which current evidence suggests is highly context specific, and likely to be higher among less diverse communities [3, 6, 48–50]. Furthermore, the health and economic circumstances of social housing residents suggests that they might live in more precarious situations with greater financial insecurity which could impact on their sense of social cohesion and health, meaning that they are an important population with which to conduct social cohesion research. These insecure circumstances might explain the lack of associations between health-related quality of life and social cohesion identified in the present study, indicating that such circumstances should be addressed before social cohesion can be developed and health improves. The sample included residents who had been in their home for only a few months and others for over a decade, which has been thought to be a determinant of social cohesion, although no clear association between social cohesion and tenure was observed (Table 2).

The relatively small sample size is a limitation of the study, limiting the level of statistical analysis that it was reasonable to undertake. However, having fewer participants enabled the collection of a breadth of data, which was necessary for a project with broad objectives like the Smartline project [21]. Although, missing data within the study was quite low, the sample are quite distinct from the local population, especially in terms of age and gender mix, limiting the external validity of the findings. Members of the public with higher sense of social cohesion may be more likely to volunteer to participate in a project researching community as well as individual health. Filling missing values in the social cohesion score and SWEMWBS with the mean of the other responses may not have been the most appropriate method for filling these missing values. However, this was only applied to eleven social cohesion scores and one SWEMWBS score and is therefore unlikely to have significantly biased the results. The SF-12v2 data from Smartline has been compared with the Welsh Health Survey as this was the only national health survey in the UK to collect SF-12v2 data, and, whilst this may be seen as a potential limitation, both Wales and Cornwall are more geographically and historically similar than some regions of England. The evaluation undertaken by White et al. [51] also took place in an area of south Wales, and area with a similar mining history to Cornwall, which supports the comparisons which have been made, particularly given the use of the same instrument to measure social cohesion.

We found that unlike in larger and national studies, in this homogenous cohort of social housing residents social cohesion alone was not sufficient to impact on health or vice versa. The implication of this is that social cohesion needs to be studied within specific community contexts, where there is a recognised attribute around which the community might cohere. This is a challenge when undertaking large national surveys into social cohesion, but it may be possible to better understand the form and function of social cohesion by using either geography or some other reported characteristics which might bring people together (e.g. participation in sports or hobbies) to cluster responses. Furthermore, longitudinal research is required in order to explore the direction of causality between social cohesion and health and wellbeing. The levels of social cohesion reported by any community are an indicator of a number of potentially complex and dynamic factors. These factors might include; the history and physical environment of the community, the characteristics of the individual members, any interventions currently attempting to bring individuals together, and any sudden shocks or external circumstances such as a crime or political change. Future studies should try to capture data on these individual and environmental factors to inform the interpretation of their findings.

## Conclusions

Whilst differences in health outcomes were evident among the social housing residents participating in the Smartline project, social cohesion was only found to be significantly associated with mental wellbeing not physical or mental health-related quality of life. This supports our initial hypothesis that the associations between social cohesion and health previously identified in large national surveys would not be identified among the Smartline cohort. Additionally, social cohesion was not found to be significantly associated with the amount of time someone had lived in their home. Being social housing residents may have provided the participants with additional opportunities to develop a sense of community, relieved some financial pressures and fostered social norms which could alter the effect of social cohesion. This may suggest that while it is possible to affect social cohesion to some degree, cohesiveness alone is not sufficient to overcome inequalities, and factors such as sense of safety or a sense of control may be more influential determinants of health [4, 11–13]. Additional qualitative and quantitative research is needed to explore the form and function of social cohesion within participant defined communities, especially with those privately renting or owning their own homes. Given the current interest in asset-based approaches to support health and wellbeing, we suggest that additional studies to explore the causal relationships involving social cohesion are needed before interventions which attempt to alter social cohesion to improve health are developed [13].

## Supplementary information

**Additional file 1.** Sensitivity analysis. Three tables providing details of the sensitivity analysis undertaken of the results presented in Tables 3, 4 and 5.

## Data Availability

An anonymised version of the dataset analysed during the current study (excluding PCS and MCS) is available upon request from the Smartline project repository: https://www.smartline.org.uk/data/

## Abbreviations

95% CI: 95% confidence interval
Coef: Regression coefficient
IMD 2015: Index of Multiple Deprivation 2015 [41]
MCS: SF-12v2 Health Survey mental component summary [38]
PCS: SF-12v2 Health Survey physical component summary [38]
RUC11: Rural Urban classification [40]
SC: Social cohesion
SWEMWBS: Short Warwick Edinburgh Mental Wellbeing Scale [32]
