# Supplementary material for: Social cohesion, mental wellbeing and health-related quality of life among a cohort of social housing residents in Cornwall: a cross sectional study
Source: BMC Public Health. 2020 Jun 22;20:985. doi: 10.1186/s12889-020-09078-6 (PMC7310403; doi:10.1186/s12889-020-09078-6)
Supplement: Supplementary file 1 — Additional file 1. Sensitivity analysis. Three tables providing details of the sensitivity analysis undertaken of the results presented in Tables 3, 4 and 5. [file 12889_2020_9078_MOESM1_ESM.pdf]

Smartline Project

Social cohesion, mental wellbeing and health-related quality of life among a cohort of social housing residents in Cornwall: a cross-sectional study – Additional file – Sensitivity analysis

Table S1 – Adjusted regression of mental wellbeing (Short Warwick-Edinburgh Mental Wellbeing Scale, SWEMWBS) additionally adjusting for ethnicity and duration of residence (n=192) [34]

		Unadjusted		Adjusted for SC	
		Coef	95% CI	Coef	95% CI
Intercept		**17.75**	**12.42 to 23.07**	**12.99**	**7.11 to 18.87**
Age	Years	**0.08**	**<0.01 to 0.16**	0.08	<-0.01 to 0.15
Gender	Female	ref		ref	
	Male	0.16	-1.51 to 1.85	0.42	-1.22 to 2.05
Ethnicity	White	ref		ref	
	Other	-4.85	-10.14 to 0.45	-5.24	-10.39 to -0.09
National identity	Cornish	ref		ref	
	British	0.70	-1.07 to 2.46	0.44	-1.28 to 2.16
	Other	2.36	0.23 to 4.48	1.92	-0.16 to 4.00
IMD 2015	1st decile (most)	ref		ref	
	2nd decile	-2.35	-4.91 to 0.21	-2.29	-4.78 to 0.19
	3rd decile	-1.62	-4.44 to 1.19	-1.17	-3.92 to 1.58
	4th decile	-2.13	-4.30 to 0.04	-1.79	-3.91 to 0.32
	5th decile	-	-	-	-
	6th decile	-	-	-	-
	7th decile	-	-	-	-
	8th decile	-	-	-	-
	9th decile	-	-	-	-
	10th decile (least)	-	-	-	-
Rurality	Urban city and town	ref		ref	
	Other	0.64	-2.73 to 4.01	0.88	-2.40 to 4.16
Education	Primary and secondary	ref		ref	
	Further	-1.63	-3.53 to 0.28	-1.58	-3.43 to 0.28
	Higher	-0.07	-3.90 to 3.75	-0.12	-3.83 to 3.60
Employment	In work	1.90	-0.25 to 4.05	1.64	-0.45 to 3.74
	Education or training	1.05	-4.57 to 6.66	0.09	-5.39 to 5.58
	Retired	2.06	-0.35 to 4.47	1.64	-0.71 to 3.99
	Not in work	ref		ref	
Household size	1	**ref**		ref	
	2	**1.24**	**-0.69 to 3.18**	0.94	-0.95 to 2.82
	3	**3.45**	**0.72 to 6.18**	2.55	-0.15 to 5.25
	4	**1.83**	**-1.45 to 5.10**	1.08	-2.13 to 4.29
	5+	**5.53**	**1.55 to 9.51**	4.76	0.86 to 8.65
Duration of residence	<1 year	0.47	-2.36 to 3.30	0.27	-2.48 to 3.03
	1-3 years	1.51	-0.70 to 3.71	0.94	-1.22 to 3.11
	4-6 years	-0.46	-2.76 to 1.84	-0.51	-2.75 to 1.72
	7-9 years	-1.12	-4.06 to 1.82	-1.21	-4.07 to 1.65
	≥10 years	ref		ref	
Social cohesion		-	-	**0.22**	**0.09 to 0.35**

IMD 2015; Index of Multiple Deprivation 2015 [32], RUC 2011; Rural Urban Classification 2011 [33], SC; social cohesion

Likelihood ratio test p= 0.0004, Adjusted r^2 unadjusted; 0.1300, adjusted; 0.1797

Values in bold indicate statistically significant (p<0.05) variables

Table S2 – Adjusted regression of physical health-related quality of life (SF-12v2 Health Survey Physical component summary) additionally adjusting for ethnicity and duration of residence (n=192) [35, 36]

		Unadjusted		Adjusted for SC	
		Coef	95% CI	Coef	95% CI
Intercept		41.43	28.38 to 54.48	41.88	26.99 to 56.77
Age	Years	-0.11	-0.31 to 0.09	-0.11	-0.31 to 0.09
Gender	Female	ref		ref	
	Male	-1.35	-5.46 to 2.77	-1.37	-5.52 to 2.78
Ethnicity	White	ref		ref	
	Other	-1.85	-14.83 to 11.14	-1.81	-14.85 to 11.23
National identity	Cornish	ref		ref	
	British	-2.04	-6.37 to 2.30	-2.01	-6.38 to 2.35
	Other	-0.13	-5.33 to 5.08	-0.09	-5.35 to 5.18
IMD 2015	1st decile (most)	ref		ref	
	2nd decile	1.64	-4.64 to 7.91	1.63	-4.66 to 7.92
	3rd decile	3.45	-3.46 to 10.36	3.41	-3.55 to 10.37
	4th decile	0.08	-5.24 to 5.41	0.05	-5.31 to 5.41
	5th decile	-	-	-	-
	6th decile	-	-	-	-
	7th decile	-	-	-	-
	8th decile	-	-	-	-
	9th decile	-	-	-	-
	10th decile (least)	-	-	-	-
Rurality	Urban city and town	ref		ref	
	Other	4.46	-3.80 to 12.73	4.44	-3.86 to 12.74
Education	Primary and secondary	ref		ref	
	Further	3.98	-0.70 to 8.65	3.97	-0.72 to 8.66
	Higher	4.15	-5.22 to 13.52	4.15	-5.24 to 13.55
Employment	In work	**7.89**	**2.62 to 13.16**	**7.91**	**2.61 to 13.21**
	Education or training	**8.77**	**-5.00 to 22.53**	**8.86**	**-5.02 to 22.74**
	Retired	**1.61**	**-4.30 to 7.51**	**1.65**	**-4.31 to 7.60**
	Not in work	**ref**		**ref**	
Household size	1	ref		ref	
	2	0.77	-3.97 to 5.50	0.80	-3.98 to 5.57
	3	4.19	-2.50 to 10.87	4.27	-2.57 to 11.11
	4	0.48	-7.55 to 8.51	0.55	-7.57 to 8.68
	5+	12.26	2.50 to 22.03	12.34	2.47 to 22.20
Duration of residence	<1 year	2.38	-4.56 to 9.33	2.40	-4.57 to 9.37
	1-3 years	-0.50	-5.90 to 4.90	-0.45	-5.93 to 5.03
	4-6 years	-1.11	-6.76 to 4.53	-1.11	-6.77 to 4.55
	7-9 years	3.96	-3.25 to 11.17	3.96	-3.27 to 11.20
	≥10 years	ref		ref	
Social cohesion		-	-	-0.02	-0.35 to 0.31

IMD 2015; Index of Multiple Deprivation 2015 [32], RUC 2011; Rural Urban Classification 2011 [33], SC; social cohesion

Likelihood ratio test p= 0.8944, Adjusted r^2 unadjusted; 0.1493, adjusted; 0.1443

Values in bold indicate statistically significant (p<0.05) variables

Table S3 – Adjusted regression of mental health-related quality of life (SF-12v2 Health Survey Mental component summary) additionally adjusting for ethnicity and duration of residence (n=192) [35, 36]

		Unadjusted		Adjusted for SC	
		Coef	95% CI	Coef	95% CI
Intercept		**38.38**	**24.34 to 52.43**	**31.83**	**15.93 to 47.72**
Age	Years	0.11	-0.10 to 0.32	0.09	-0.12 to 0.30
Gender	Female	ref		ref	
	Male	3.09	-1.34 to 7.52	3.44	-0.99 to 7.86
Ethnicity	White	ref		ref	
	Other	5.80	-8.18 to 19.77	5.25	-8.66 to 19.17
National identity	Cornish	ref		ref	
	British	2.20	-2.47 to 6.87	1.85	-2.81 to 6.51
	Other	4.30	-1.30 to 9.90	3.70	-1.92 to 9.31
IMD 2015	1st decile (most)	ref		ref	
	2nd decile	-6.70	-13.46 to 0.05	-6.63	-13.34 to 0.09
	3rd decile	-3.82	-11.26 to 3.61	-3.20	-10.63 to 4.23
	4th decile	-3.35	-9.08 to 2.38	-2.88	-8.61 to 2.84
	5th decile	-	-	-	-
	6th decile	-	-	-	-
	7th decile	-	-	-	-
	8th decile	-	-	-	-
	9th decile	-	-	-	-
	10th decile (least)	-	-	-	-
Rurality	Urban city and town	ref		ref	
	Other	-6.53	-15.43 to 2.36	-6.20	-15.06 to 2.66
Education	Primary and secondary	ref		ref	
	Further	2.04	-2.99 to 7.07	2.11	-2.89 to 7.11
	Higher	-1.85	-11.94 to 8.23	-1.91	-11.94 to 8.12
Employment	In work	**7.83**	**2.16 to 13.50**	**7.48**	**1.83 to 13.13**
	Education or training	**-2.13**	**-16.95 to 12.69**	**-3.44**	**-18.25 to 11.37**
	Retired	**6.95**	**0.60 to 13.30**	**6.37**	**0.02 to 12.72**
	Not in work	**ref**		**ref**	
Household size	1	**ref**		ref	
	2	**2.18**	**-2.92 to 7.27**	1.75	-3.34 to 6.84
	3	**3.21**	**-3.99 to 10.40**	1.97	-5.33 to 9.26
	4	**-2.02**	**-10.66 to 6.62**	-3.04	-11.72 to 5.63
	5+	**15.24**	**4.73 to 25.75**	14.18	3.65 to 24.70
Duration of residence	<1 year	-9.00	-16.47 to -1.53	-9.27	-16.71 to -1.83
	1-3 years	-0.75	-6.56 to 5.06	-1.53	-7.38 to 4.32
	4-6 years	-3.08	-9.16 to 2.99	-3.15	-9.19 to 2.89
	7-9 years	-6.09	-13.85 to 1.67	-6.21	-13.93 to 1.50
	≥10 years	ref		ref	
Social cohesion		-	-	0.30	-0.05 to 0.65

IMD 2015; Index of Multiple Deprivation 2015 [32], RUC 2011; Rural Urban Classification 2011 [33], SC; social cohesion

Likelihood ratio test p= 0.0691, Adjusted r^2 unadjusted; 0.0972, adjusted; 0.1073

Values in bold indicate statistically significant (p<0.05) variables
